# Centipede Venom: A Potential Source of Ion Channel Modulators

**DOI:** 10.3390/ijms23137105

**Published:** 2022-06-26

**Authors:** Anna Luo, Aili Wang, Peter Muiruri Kamau, Ren Lai, Lei Luo

**Affiliations:** 1 Center for Evolution and Conservation Biology, Southern Marine Science and Engineering Guangdong Laboratory (Guangzhou), Guangzhou 511458, China; luoanna@mail.kiz.ac.cn (A.L.); allie612@gmlab.ac.cn (A.W.); 2 Key Laboratory of Animal Models and Human Disease Mechanisms of the Chinese Academy of Sciences/Key Laboratory of Bioactive Peptides of Yunnan Province, KIZ-CUHK Joint Laboratory of Bioresources and Molecular Research in Common Diseases, National Resource Center for Non-Human Primates, Kunming Primate Research Center, National Research Facility for Phenotypic & Genetic Analysis of Model Animals (Primate Facility), Sino-African Joint Research Center, and Engineering Laboratory of Peptides, Kunming Institute of Zoology, Kunming 650107, China; peter@mail.kiz.ac.cn; 3 University of Chinese Academy of Sciences, Beijing 100049, China

**Keywords:** centipede, toxin, SsTx, RhTx, ion channel

## Abstract

Centipedes are one of the most ancient and successful living venomous animals. They have evolved spooky venoms to deter predators or hunt prey, and are widely distributed throughout the world besides Antarctica. Neurotoxins are the most important virulence factor affecting the function of the nervous system. Ion channels and receptors expressed in the nervous system, including Na_V_, K_V_, Ca_V_, and TRP families, are the major targets of peptide neurotoxins. Insight into the mechanism of neurotoxins acting on ion channels contributes to our understanding of the function of both channels and centipede venoms. Meanwhile, the novel structure and selective activities give them the enormous potential to be modified and exploited as research tools and biological drugs. Here, we review the centipede venom peptides that act on ion channels.

## 1. Introduction

Throughout human history, venomous creatures such as snakes, scorpions, centipedes, caterpillars, and poisonous frogs have existed in the human living environment and civilization. In most ancient cultures, venomous creatures were either deified and associated with the divine, or vilified as inflictors of pain and distress due to their mysterious and destructive power. As one of the most ancient and successful venomous predators, centipede venom is an excellent model for insight into venoms’ functional and molecular evolution. Centipedes originated around 440 million years ago with almost worldwide distribution except for Antarctica, and comprise approximately 3300~3500 extant species that are divided into five categories: *Craterostigmomorpha*, *Geophilomorpha*, *Lithobiomorpha*, *Scutigeromorpha*, and *Scolopendromorpha*, making them one of the oldest and the richest lineages of living venomous terrestrial predators [1].

For centuries, centipedes have been frequently involved in human accidents [1,2]. Most reported cases of envenomation caused by centipede stings and bites often displayed symptoms that include localized pain and necrosis, paresthesia, lethargy, headache, dizziness, and nausea, and a small percentage of cases showed more grievous symptoms including anaphylaxis, hemorrhage, and even hematuria [2,3,4,5]. The complexity and diversity of symptoms of centipede bites indicate that centipede venom possesses a rich native source with functional diversity. Based on the highly developed technology, it is verified that centipede venoms are comprised of a variety of bioactive proteins/peptides with multiple disulfide bonds, which give excellent chemical, heat, and biological stability to peptide toxins [6,7]. Among them, centipede peptide toxins are classified into 31 families phylogenetically and exhibit different biomedical properties pharmacologically, including ion channel activity, antibacterial activity, platelet aggregation activity, antithrombotic activity, phospholipase A2 activity, and trypsin-inhibiting activity [7,8,9,10,11]. 

From the view of survival strategy and clinical features, neurotoxins affecting the nervous system might play an important role in centipede envenomation [6,8,12,13,14,15]. In the evolution of centipedes, the diversity of neurotoxins in structure and function might be the result of adaptive traits as a hunting device. On the other hand, in the development of drug and clinical diagnosis, further insight into the mechanism of neurotoxic manifestations is required for the development of engineered toxin-based applications. Promoting insight into compositions and functions of centipede venom, in turn, positively contributes to discovering novel molecules or new tools for ion channels. Here, we review and summarize the reported centipede peptides acting on ion channels, including voltage-gated sodium (Na_V_) channels, voltage-gated potassium (K_v_) channels, voltage-gated calcium (Ca_V_) channels, and transient receptor potential cation channels (TRP).

## 2. Diversity of Centipede Venoms

For different strategies and purposes, venomous animals have evolved complex venom systems that have independently evolved more than one hundred times from non-toxic ancestral peptides [16]. According to a venomous lineages survey, venom’s primary functions are promoting feeding, immobilizing, or killing prey, and defending against potential predators, with offspring and antimicrobial attributes contingently [16]. Rynald et al. showed that centipede gene families that encode peptides toxins had horizontally transferred between centipedes, bacteria, and fungi repeatedly throughout their evolution [17]. Furthermore, five gene families from bacteria and fungi transferred to the centipede, and three protein families involved in pore-formation and enzymatic action from bacteria turned into virulence factors in centipede venom [17]. These findings suggest that multiple horizontal gene transfers (HGTs) have contributed to centipede venoms’ recycling of xenogenous proteins as activity components for predation. Therefore, the research established that the centipede is the only known venomous creature that contains multiple gene families originating from horizontal gene transfer to encode peptide toxins.

Most proteins in centipede venoms are homologous with that of other venomous animals, while some centipede peptides surprisingly bear no or few resemblances to any other characterized venomous arthropod peptide family [7]. In 2014, we identified 79 unique peptide toxins from the centipede *Scolopendra subspinipes mutilans* L. Koch by peptidomics and cDNA library, further strengthening the diversity of centipede venoms [9]. Of these, 29 were identified as neurotoxins targeting Na^+^, K^+^ and Ca^2+^ channels [7,9]. These peptide toxins were named and divided into 17 families based on sequences and cysteine arrangement. There were three novel families (SLPTX26-29) that were first discovered among filtered peptide toxins from centipedes because of the special cysteine partner. Most mature peptides of centipede toxins contain four or six cysteines in the same arrangement as other animal toxins, while some centipede toxins show an uncommon number of cysteines; for instance, U-SLPTX28-Ssm1a with three cysteines, U-SLPTX15-Ssm2a with five cysteines and U-SLPTX12-Ssm1a with seven cysteines [9]. Interestingly, two previously unknown novel cysteine frameworks “C-C-C-CCC” in U-SLPTX8-Ssm1a,1b,1c and “C-C-C-C-CC-CC” in U-SLPTX27-Ssm1a, 2a, 3a were first discovered as an original motif in peptide toxins that offers qualified support for the diversity of centipede toxin(Table 1) [9].

Furthermore, similar results were also found by Rokyta et al. on *Scolopendra viridis* [18]. According to proteomic-driven annotation, the final transcripts from multiple individual units produced 520 unique protein-coding transcripts classified into two types proteomically: toxins and nontoxins [10,18]. The most dominant toxins in the venom of *S. viridis* contain peptides acting on Ca^2+^ and K^+^ channels, allergens, metalloproteases, and β pore-forming toxins [10]. Fourteen members of the Scoloptoxins (SLPTXs) family inhibit either potassium or calcium channels. Seven β-PFTx transcripts contain an 18~20 amino acid signal peptide characterized with a β-complex domain, such as aerolysin toxins. Four metalloproteases (MPs) of the M12A family involved in modification after translation mediating activation of other peptide toxins. Two centiCAP2 transcripts and other proteins/peptides including chitinase, venom hyaluronidase (HYAL) and proteins contain an LDLA (low-density lipoprotein receptor Class A repeat) domain [7,10,18]. *S. viridis* (FL) has been compared with previously published *S. viridis* (MO) in transcriptomes and proteomes, which unexpectedly exhibit extreme levels of variation of *S. viridis* [10,18].

In addition, the work of Gunnar S. Nystrom et al. filled a gap in sexually dimorphic traits in centipede venom that were reported in invertebrate venoms, such as scorpions and spiders. They identified 47 toxins and 717 nontoxic transcripts by RP-HPLC, venom proteomes, and venom-gland transcriptomes on both female and male eastern bark centipedes (*Hemiscolopendra marginata*) and confirmed it as the first sample of centipede venom variation based on sex [19]. According to the results of LC-MS/MS confirmation analyses, there is no striking sex-based difference in the contents of the eight identified members of the CAP2 and CAP3 in *H. marginata* [19]. At the same time, a significantly higher GGT (γ-glutamyl transferase family) proteomic abundance was detected in female *H. marginata* than in male venom, which has been reported to enhance platelet aggregation and hemolysis [19]. On the other side, SLPTXs in male venom were expressed distinctly and significantly higher than the female with a proportion of 4.3% and 26.1% by proteomics [19]. Overall, the identified ion channel-targeting peptides had the highest expression in the male venom, while in the female venom were γ-glutamyl transferases and CAPs [19]. Combined with feeding ecology and behavior information, the differential expression of toxins may contribute to understanding sex-based transcriptional variation.

Overall, centipede venoms have been intensively studied over the last ten years. These studies take us a step further in understanding how centipedes evolved various neurotoxins to target receptors and take advantage of this system to paralyze and kill prey or defend against predators. Meanwhile, it is important to study the natural effects of venom components for exploiting them as drugs.

**Table 1:** Centipede toxins acting on ion channels.

Peptide Toxin	Organism	Number of Residues	PDB	Ion Channels Activity
μ-SLPTX-Ssm6a (μ-SLPTX3-Ssm6a)	*Scolopendra mutilans*	46	2MUN\2MZ4	Na_V_1.7, IC_50_ of 25 nM
μ-SLPTX-Ssm1a	*Scolopendra subspinipes*	32	-	TTX-S Na_V_ channel, IC_50_ of 9 nM
ω-SLPTX-Ssm1a	*Scolopendra subspinipes*	83	-	activator of Cav channels in DRG
ω-SLPTX-Ssm2a	*Scolopendra subspinipes*	54	-	Ca_V_ channels in DRG, IC_50_ of 1590 nM
SsmTx-I [20]	*Scolopendra subspinipes mutilans*	36		K^+^ channels in DRG, IC_50_ of 200 nM; K_V_2.1, IC_50_ of 41.7 nM; No effect on other K^+^ channels
SsTx	*Scolopendra mutilans*	53	5X0S	K_V_1.3, IC_50_ of 5.26 μM
SsdTx1-3	*Scolopendra subspinipes dehaani*.	53		blocking hKir6.2 with a K_d_ of 278\260\281 nM
SpTx1	*Scolopendra subspinipes dehaani*.	54		Inhibiting hKir6.2 with a K_d_ of 8.5 nM
SsTx-4	*S.subspinipes mutilans*	53		Kir6.2, IC_50_ of 42.5 nM at 140 mV and 75.4 nM at 40 mV; Kir1.1, IC_50_ of 89.2 nM at 140 mV and 209.7 nM at 40 mV; Kir4.1, 360.1 nM at 140 mV and 6.2 μM at 40 mV
RhTx	*Scolopendra mutilans*	27	2MVA	TRPV1, EC_50_ of 521.5 nM
RhTx2	*Scolopendra mutilans*	31	3J5P\3J5Q	TRPV1, EC_50_ of 38.35 μM

## 3. Centipede Venom Components Acting on Na_V_ Channel

**Figure 1:**
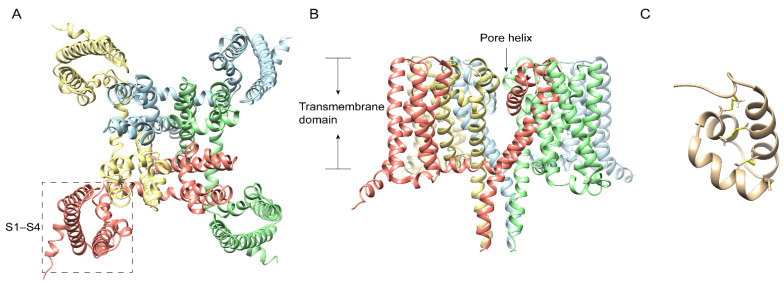
Ribbon diagram of human Na_V_1.7 atomic model (PDB id: 5EK0) with each of the four subunits color-coded, showing views from the bottom (**A**) and side (**B**). The voltage-sensing domain is labeled with a dashed box. The membrane-spanning helices and different subunits of Na_V_1.7 channel are indicated. (**C**): μ-SLPTX-Ssm6a from the Chinese red-headed centipede *Scolopendra subspinipes*. PDB id: 2MUN.

Pain, the most classic symptom of centipede bites, is a crucial adaptive reaction that helps limit the degree of exposure to potential hazards or threatening events. Thus, through the study of nociception with centipedes, it is possible to develop the next generation of analgesics. Na_V_ channels are vital transmembrane proteins mainly expressed in excitable cells that mediate rapid depolarization and participate in nociceptor responses accordingly [21]. Compared with only one subtype of Na_V_ channel in insects, humans express nine different Na_V_ channel subtypes, registered as Na_V_1.1 to Na_V_1.9 [22,23]. Therein, the Na_V_1.7 channel (Figure 1A,B) was reported as a promising analgesic target. We purified and identified μ-SLPTX-Ssm6a (Figure 1C), a unique peptide with 46 residues, from the venom of the Chinese red-headed centipede *Scolopendra subspinipes* [8], which was reported to strongly and selectively inhibit Na_V_1.7 with an IC_50_ of 25 nM and act as a more effective analgesic than morphine in rodent pain models without side effects [8]. The specificity on Na_V_1.7 gives μ-SLPTX-Ssm6a therapeutic potential. At the same time, the unique 3D-fold of one α-helical, two to three β-strands, and the disulfide linkage pattern give Ssm6a a high level of resistance to proteases and thermal stability for long-term analgesic efficacy [8]. Furthermore, μ-SLPTX-Ssm1a selectively inhibited TTX-S Na_V_ channel currents in rat DRGs with an IC_50_ of ~9 nM with no visible effect on TTX-R Na_V_ currents [8]. From the biological point of view, centipedes are likely to paralyze rapidly and ultimately kill insect prey by a rapid blockage of insect Na_V_ channels, similar to blocks on human Na_V_1.7 within seconds.

For the great selectivity and attractive bioactivity of μ-SLPTX-Ssm6a, Chuan Wang et al. developed a new protein scaffold fusion method to purify Ssm6a and maintain the selectivity and potency at the same time. With a protein scaffold transformed from human protein, they designed, expressed, and purified the fusion protein SP-TOX. Similar to Ssm6a, SP-TOX inhibited TTX-S current by 93.6% with little effect on TTX-R current at 1 μM [24]. On formalin-induced inflammatory pain, SP-TOX significantly relieved the inflammatory pain in a Phase II study with a concentration as low as 0.02 nM, which is 30~50% more potent than morphine at 0.035 nM and 0.35 nM [24].

## 4. Centipede Venom Components Acting on Cav Channel

**Figure 2:**
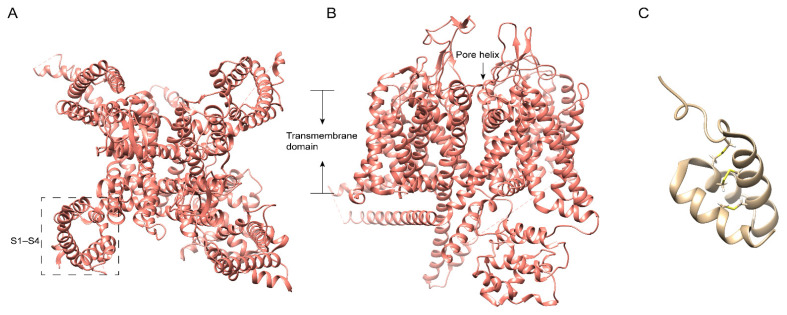
Structures of N-type voltage-gated calcium channels (Cav2.2) and centipede venoms. Ribbon diagram of the Cav2.2 atomic model (PDB id: TMIY) showing views from the bottom (**A**) and side (**B**). The voltage-sensing domain is labeled with the dashed box. The membrane-spanning helices of the Cav2.2 channel are indicated. (**C**): k-Ssm1a from *Scolopendra subspinipes*. PDB id: 2M35.

The voltage-gated calcium channel (Ca_V_) is composed of α1 subunit-containing pore-forming and voltage-sensing domains, and several α2δ, β, and γ regulatory subunit isoforms [25]. According to the voltage ranges required for activation, Cav superfamily 3 T-type (Ca_V_3.1–3.3) channels are denoted as low-threshold channels, while superfamilies 1 and 2 are high-threshold channels, including L-type (Ca_V_1.1/4), N-type (Ca_V_2.2), P/Q-type (Ca_V_2.1), and R-type (Ca_V_2.3) channels [25]. The N-type Ca_V_2.2 (Figure 2A) is mainly expressed at the nerve terminal throughout the CNS (central nervous system) and PNS (peripheral nervous system), wherein a common pathway downstream from various receptors mediates pain responses; thus, inhibition of Ca_V_2.2 might mediate analgesia [26]. Meanwhile, Ca_V_2.2 knock-out mice showed high resistance to neuropathic pain and insensitivity to visceral pain induced by formalin, with normal CNS and physical performance [27,28]. Using peptidomics, transcriptome analysis and electrophysiological assays, 26 peptides in ten neurotoxin-like groups were identified from the venom of *Scolopendra subspinipes mutilans* [7]. Interestingly, two of them showed activity at Ca_V_ channels. ω-SLPTX-Ssm1a (Figure 2C), an 83 residue peptide with 7 cysteines, activates both vertebrate and invertebrate Cav channels as Ca^2+^ currents in rat DRGs with the application of ω-SLPTX-Ssm1a showed an increase of 70, and 120% at 1 and 10 μM, respectively [7], while ω-SLPTX-Ssm2a (Figure 2B) with 54 residues inhibits Ca_V_ channels expressed in DRG with an IC_50_ of 1.6 μM [7]. According to the result of BLAST, ω-SLPTX-Ssm2a shares similar disulfide frameworks with several lycotoxins identified in the araneomorph spider *Lycosa singoriensis*, while the N- and C-termini of them are profoundly distinct [7]. Except for ω-SLPTX-Ssm2a, the venom peptides from *Scolopendra subspinipes mutilans* L. Koch show no similarity to any identified protein families from any venomous creature [7].

## 5. Centipede Venom Components Acting on the K_V_ Channel

**Figure 3:**
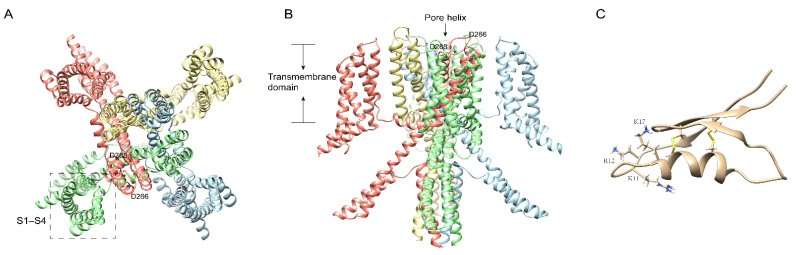
The cryo-electron microscopy (cryo-EM) structure of human KCNQ4 and SsTx. Ribbon diagram of KCNQ4 atomic model (PDB id: 7VNP) with each of the four identical subunits color-coded, showing views from the bottom (**A**) and side (**B**). The key residues for the interaction with SsTx are labeled. The membrane-spanning helices and different subunits of KCNQ4 channel are indicated. (**C**): SsTx from *Scolopendra morsitans*. PDB id: 5X0S.

Potassium (K^+^) channels are known to be the most varied channels. According to the structure of ɑ-subunits, potassium channels are further divided into four categories: voltage-gated potassium (K_v_) channels, calcium-activated potassium (K_Ca_) channels, inwardly rectifying potassium (K_ir_) channels, and two-pore potassium (K2P) channels [29,30,31,32]. K_V_ channels selectively mediate the potassium transmembrane transportation induced by voltage or other physiological mediators. They are found in almost all cell types and involved in various vital activities, including the release of neurotransmitters and hormones and controlling membrane properties [30]. For example, ATP-sensitive K^+^ (KATP) channels are expressed in pancreatic β cells that secrete insulin and is regarded as the regulator between the level of blood glucose and insulin secretion [33]. The K_V_1.3 channel is highly expressed in immune cells and is considered an alternative target in treating AID (autoimmune disease) [34,35]. In 2018, we first identified Ssm Spooky Toxin (SsTx)(Figure 3C) from *Scolopendra mutilans*, which exhibited lethal toxicity in hematological and respiratory systems by potently inhibiting KCNQ (voltage-gated potassium channel family 7) channels (Figure 3A,B) [6,36]. Furthermore, SsTx also inhibits cytokine generation by specifically acting on the K_V_1.3 channel in T cells [6]. Therefore, it is considered a crucial virulence factor in red-headed centipedes’ defense and predation due to its efficient disruption of cardiovascular, respiratory, muscular, and nervous systems. Interestingly, SsTx also exhibited potent inhibitory activity on the K_V_1.3 channel with an IC_50_ of 5.26 μM, which would amplify the disruptive effect of blocking KCNQ channels [6]. Mechanically, we found that residue K11\R12 (Figure 3C) in SsTx targeting the outer pore region and the selectivity filter of K_V_1.3 and K_V_7.4 provides the key sites that bind the toxin exclusively to channels [6]. Besides the great potential of being developed and utilized, SsTx is also an interesting toxin component to understand how a single toxin develops different intraspecific and interspecific antagonistic interactions. We found that centipedes deterred conspecifics with short-period, reversible, and not fatal envenomation by inhibiting the Shal (centipede K_V_ channel subtypes) channel [37]. The Shal channel, consisting of six transmembrane helices and a pore domain, is expressed in the DUM (dorsal unpaired median) neurons and heart tube, playing an essential role in the centipede’s circulatory system. SsTx specifically inhibits currents from the Shal channel with an IC_50_ from 0.1 to 0.3 M [37]. With thermodynamic mutant cycle analysis and molecular docking, pore-blocking inhibition is the effect of salt bridge bonding between E351 on Shal and K17 (Figure 3C) on SsTx [37]. Based on an acknowledged view that most neurotoxins are ineffective on their own receptors, paralysis caused by SsTx from conspecifics provided an example to understand intraspecific deterrence.

Further, a similar family of peptides that inhibit hK_ATP_ were identified from the venom of *Scolopendra subspinipes dehaani*. Functionally, four of these proteins (SsdTx1-3 and SsTx) inhibit hK_ATP_ channels by blocking hKir6.2 with a K_d_ of <300 nM [38]. In addition, SsTx-4 also showed inhibitory activities on Kir1.1 and Kir4.1 channels, while other Kirs were insensitive to SsTx-4 application [39]. SsTx-4 is a peptide with 53 residues containing four cysteines, which shows high homology to SsTx and SsdTx1-3. Therefore, it is reasonable that nSsTx-4 (natively purified from the venom) was found to inhibit the Kir6.2 currents with an IC_50_ of 42.5 nM at 140 mV [39]. Meanwhile, SsTx-4 also inhibited the currents of Kir1.1 and Kir4.1 with an IC_50_ of 89 nM and 360 nM at 140 mV, respectively. This work confirmed that SsTx-4 is the first known peptide toxin antagonistic to the Kir4.1 channel [39]. To confirm the activity and molecular mechanisms of SsTx-4, they expressed it with an added glycine residue at the N terminus that was proven not to affect its activity [39]. They found that both nSsTx-4 and rSsTx-4 (recombinantly expressed toxin) reduced inward currents more potently than outward currents, especially for Kir4.1. Further, the M137 residue at the Kir channel’s M1-M2 domain might be the key molecule in the Kir6.2 channel for interacting with SsTx-4 [39]. On the other hand, the K13 and F14 in the p-p segment of SsTx-4 were identified as the critical residues involved in binding to the Kir6.2 channel, for mutations of these sites profoundly impaired the toxin’s effectiveness [39]. At the same time, these two residues are also critical sites in SsTx-4 for binding to the Kir1.1. Furthermore, it is interesting that K11 is merely functional for SsTx-4 targeting Kir1.1, and F43 is the key residue on the toxin only against Kir6.2 but not for Kir1.1. Meanwhile, F44A mutation induced an approximate impairment of SsTx’s affinity for both channels [39]. The binding with Kir4.1, K13, F43, and F44 in SsTx-4 were confirmed as the determinants for mutations of these impaired toxin affinities and increased IC_50_ of Kir4.1 [39]. These works on SsTx contribute to our understanding of the function and molecular mechanisms of centipede venom and promote the development and application of peptide toxins.

## 6. Centipede Venom Components Acting on TRP and Other Channels

**Figure 4:**
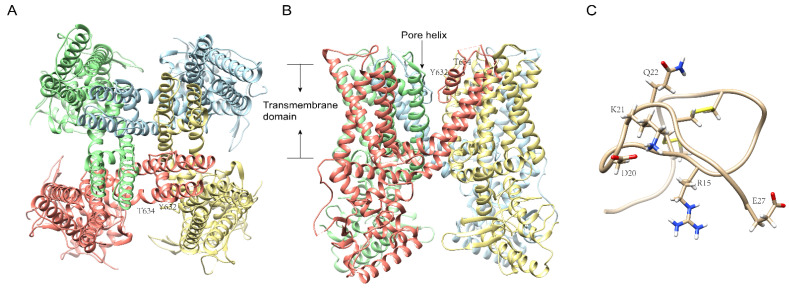
The cryo-electron microscopy structure of TRPV1 and RhTx. Ribbon diagram of TRPV1 atomic model (PDB id: 7L2L) with each of the four identical subunits color-coded, showing views from the bottom (**A**) and side (**B**). The key residues for the interaction with RhTx are labeled. The membrane-spanning helices and different subunits of the TRPV1 channel are indicated. (**C**): RhTx from *Scolopendra subspinipes*. PDB id: 2MVA.

TRPV1 is a well-studied channel known as an autologous and environmental noxious stimuli receptor, for instance, high temperature above 40 °C, pH below 6.0, peptide toxins, and the most classic, capsaicin, the hot chemical in chili [40,41]. As revealed by cryo-electron microscopy and study of structural and physiological function, it is known that the TRPV1 (Figure 4A,B) receptor consists of four isologous subunits, and that every subunit contains six transmembrane helixes [42]. Structurally, the pore-forming loop between helixes 5 and 6, also called the pore helix, together with the N- and C-termini, forms a functional channel pore allowing the transmembrane transport of cations [42]. Moreover, 112 sites along the sequence have been successively found to functionally respond to various kinds of activators, inhibitors, and pore blockers [43]. Functionally, taking advantage of two invaluable ligand tools, the plant toxins capsaicin and resiniferatoxin (RTX), a potent phytotoxin activator discovered from the plants *Euphorbia resinifera* and *Einhorbia poissonii*, researchers have achieved the first cloning and characterization of TRPV1, which brought insight into the function of TRPV1 in itch, cancer, and weight loss [44]. According to research on LUAD (lung adenocarcinoma) patients, TRPV1 expression is notably up-regulated in the tumor tissues, which indicates that TRPV1 might be an alternative novel target for LUAD treatment [45]. 

We identified RhTx (Figure 4C), a peptide toxin with 27 amino acids, from the venom of the Chinese red-headed centipede and confirmed it as a selective TRPV1 activator with comparable efficacy to capsaicin and no activity on Kv2.1 and other TRPV channels [15]. Furthermore, we found that there are three key residues in TRPV1 D602 located in the filter, Y632, and T634 (Figure 4A,B) in the pore domain, respectively, that interact with four charged residues (D20, K21, Q22, and E27) and R15 (Figure 4C) in RhTx by electrostatic interactions [15]. Interestingly, RhTx activity was positively correlated with temperature in functional examinations, as rising temperatures profoundly intensify the toxin activity and even deactivate it completely at 10 °C [15]. In addition, RhTx can desensitize TRPV1′s activation by heat alone, while not affecting activation by capsaicin [15]. These findings imply that natural toxins are an ample and valuable source of tools for understanding the difference between heat-driven and ligand-driven TRPV1 activation and regulation mechanisms.

Furthermore, in 2020, we once more identified a peptide that is identical to RhTx except for four additional residues (NSKY) at the N terminus, hence named RhTx2 [46]. Analogously, RhTx2 strongly activates TRPV1 from the extracellular side, which cannot be competitively blocked by capsazepine like RhTx. However, the binding affinity of RhTx2 to TRPV1 is much lower than RhTx, with an EC_50_ of 38.35 ± 4.61 μM, which is nearly 100 times that of RhTx [46]. Furthermore, RhTx2 was found to induce significantly faster desensitization and relatively slow recovery on TRPV1 upon prolonged application [46]. With single-channel recordings, both the open probability and the single-channel conductance were reduced considerably during RhTx2-induced desensitization of TRPV1 current. According to the docking simulation results, RhTx2 might hinder the process of ion permeation by binding to a central position above the selectivity filter of the channel [46]. With RhTx and RhTx2, we were able to get insight into the functional, structural, and biophysical properties of TRPV1, which contribute to understanding TRPV1 regulation as an ion channel and a receptor in the pain pathway.

Despite the P2X receptor having been verified to be involved in a broad range of physical performance disturbances, including hypertension, bladder incontinence, chronic cough, inflammatory and immune disorders, megrim, pain, IBS (irritable bowel syndrome), epilepsy, atherosclerosis, depressive disorder, diabetes mellitus, and cancer, purinergic receptors have been seldom considered and involved in the development of exploiting novel molecules, or new tools from animal toxins that modulate ion channels [47,48,49,50,51,52,53,54,55]. Up to now, the only reported toxin that potently and selectively targets P2X3 came from the venom of a wolf spider by Grishin et al. in 2009 [56]. To further promote the quick identification of natural toxins and facilitate purine-target drug development, Leanne Stokes et al. developed quantitative high-throughput fluorescent-based screen (HTS) cell-based assays that afford a sensitive and specific method to identify and purify novel P2X inhibitors from crude venoms. Based on HTS, they screened and validated 180 crude venoms from arachnids, centipedes, hymenopterans, and cone snails analytically and pharmacologically, which has contributed a lot to the early phases of the drug discovery process [57]. During the cell-based HTS, most of the validated components that inhibit hP2X4 were derived from spider venoms [57]. However, centipede venoms and purinergic receptors remain a potential source of drugs and targets of diseases.

## 7. Conclusions 

Humans have strugglingly and progressively coexisted with venomous creatures for centuries. With technology development, people have intensively studied some well-known venomous animals, such as snakes, spiders, and frogs [58]. However, despite being frequently involved in human accidents and used in traditional medicines, centipedes and their venoms have not been fully recognized for a considerable time and there is a lack of in-depth understanding of their biochemical and pharmacological properties. With the increasing need for adequate pain control and novel drug discovery, people have turned their attention to centipede venoms and ion channels. Using a cDNA library, bioinformatic analyses like proteomics and transcriptomics and electrophysiological assays have been applied to the study of centipedes and venoms of several varietals across the world have been high-throughput screened, analyzed, and validated over the last ten years, both structurally and pharmacologically [9,10,11,17,18]. Consistent with the diverse symptoms of centipede bites and the most typical intense local pain, many new components and peptides with unique structures and original sequences have been identified, which represent multitudinous physiological activities and targets including the K_V_ channel, Na_V_ channel, Ca_V_ channel, and other channels [6,7,8,15,38]. As mentioned above, there is an interesting variety of centipedes. The venom peptide from *Scolopendra subspinipes mutilans* L. Koch exhibits no similarity to any known peptide toxin families from any venomous animal [7]. Three of them, μ-SLPTX-Ssm6a, μ-SLPTX-Ssm1a, and ω-SLPTX-Ssm2a, are potent and selective inhibitors targeting Na_V_1.7, TTX-S Na_V_ channel, and Ca_V_ channels respectively [7]. Ssm Spooky Toxin (SsTx) inhibits both KCNQ and K_V_1.3 channels, which are considered to mutually intensify the inhibitory effect [6]. Furthermore, two of them, ω-SLPTX-Ssm1a and RhTx, are selective activators of the Cav channel and TRPV1 [7,15]. Interestingly, the activity of RhTx exhibits a positive correlation with temperature and specific desensitization to TRPV1′s activation by heat [15]. All of these complicated structures and functions indicate that centipede peptide toxins with high potency and specificity have the potential to be novel research tools for ion channels and sources of biopharmaceutical candidates. With the development and application of technologies, more centipedes and their peptide toxins are expected to be identified by transcriptomics and proteomics and validated by electrophysiology in further study.
